# Subgaleal Collection: An Unusual Presentation of a Scalp Mass in a Pediatric Patient

**DOI:** 10.7759/cureus.62322

**Published:** 2024-06-13

**Authors:** Robin Okpara, Anthony Pham, Roy Jacob

**Affiliations:** 1 Department of Radiology, Texas Tech University Health Sciences Center School of Medicine, Lubbock, USA; 2 Department of Radiology, Texas Tech University Health Sciences Center, Lubbock, USA

**Keywords:** trauma, subgaleal hematoma, hair pulling, hair braiding, head injury, subgaleal collection

## Abstract

Subgaleal fluid collection is a rare phenomenon of scalp swelling among young infants and, in many cases, adolescents. As fluid accumulates in the subgaleal space, it presents as a soft, ill-defined, fluctuant, mobile swelling not limited to suture lines. This condition is associated with vacuum-assisted devices and forceps during delivery in infancy. Beyond infancy, this condition can be seen spontaneously or, most commonly, after minor head traumas. Such minor traumas that have been reported in recent years include hair pulling or hair braiding. Early recognition of this condition and its complications is essential for appropriate treatment and management. In this case report, we highlight the importance of subgaleal fluid collection being considered a differential diagnosis of headaches, particularly in children and adolescents who present with excessive hair pulling or hair braiding.

## Introduction

A collection of fluid in the subgaleal space is a manifestation often seen in neonates and infants. They are strongly associated with vacuum extraction and forceps delivery, causing hemorrhage or extradition of fluid into the disrupted tissue planes at the time of delivery [1,2]. Beyond the neonatal period, subgaleal fluid collection is rarely reported in children and usually may arise spontaneously or, more commonly, secondary to minor head trauma. Interestingly, a common method of injury seen is excessive hair pulling and hair braiding [3-6]. In many cases, excessive hair pulling and hair braiding can lead to the development of a more severe complication known as a subgaleal hematoma (SGH). Recognition of this clinical manifestation is essential for appropriate management and prevention of unnecessary interventions or workups for nonaccidental trauma in an otherwise normal infant. This case report describes a 13-year-old female patient who presented with a chief complaint of a headache and was found to have a subgaleal fluid collection around the superior parietal region of the scalp.

## Case presentation

Our patient was a 13-year-old African-American female who presented to the emergency department with a chronic, fluctuating headache over the past few weeks. She described her headache as a throbbing pain, primarily focused around the frontal and temporal regions of the head. It was exacerbated by light tugging and pulling on the scalp when combing her hair. No relieving factors nor associated symptoms of dizziness, nausea, vomiting, photophobia, neck stiffness, and altered level of consciousness were noted. The patient and her family endorse a long history of hair braiding since she was a toddler. Her family denied any history of trauma in addition to patient or family history of headaches, strokes, and seizures.

**Figure 1:**
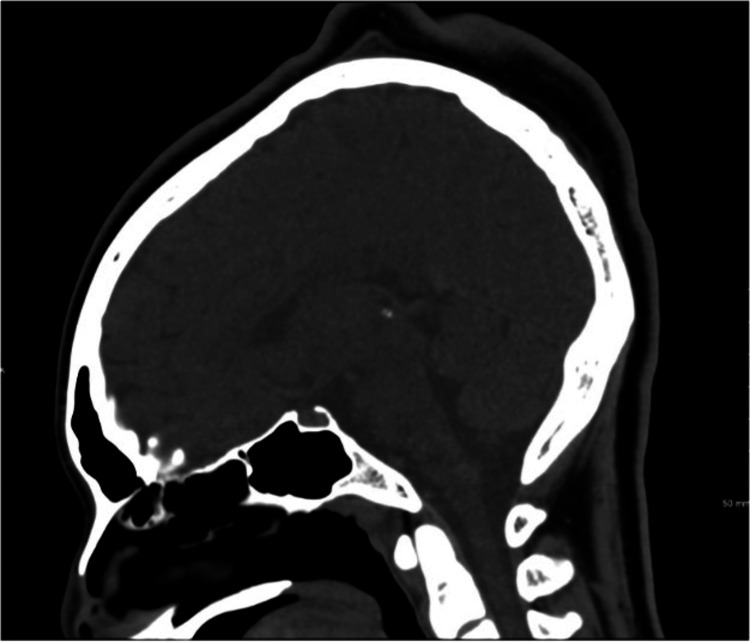
Sagittal noncontrast computed tomography of the head demonstrates an undulating contour of the scalp in the posterior parietal region

**Figure 2:**
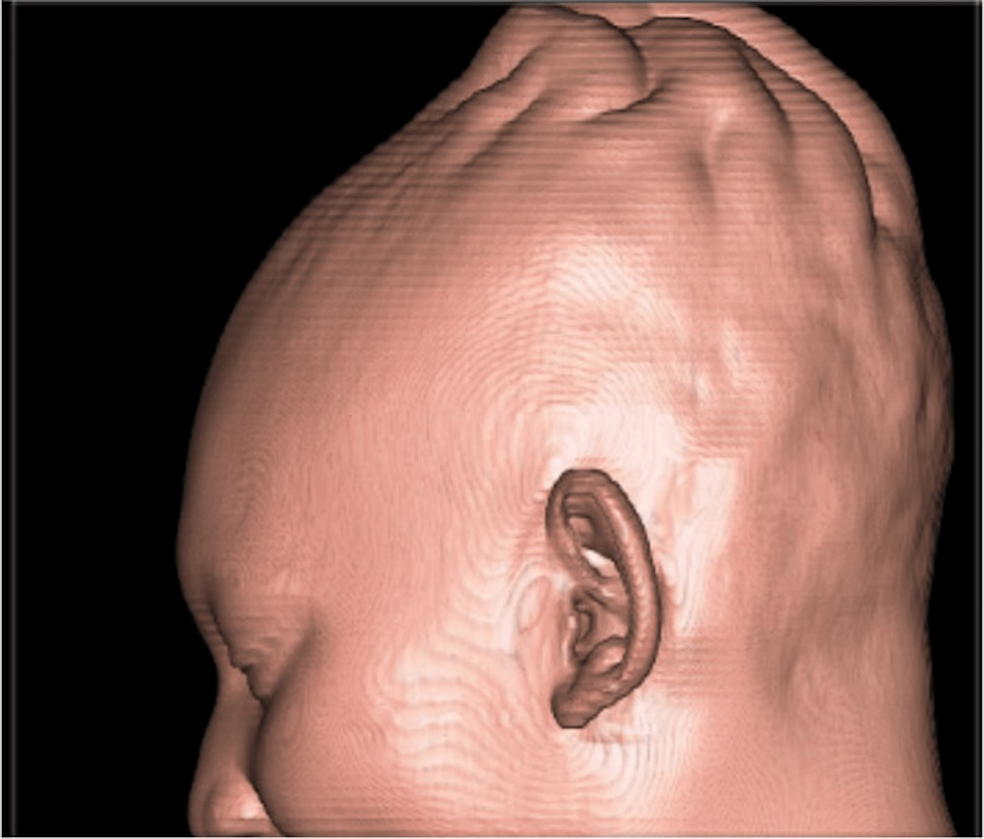
3D surface-shaded volume rendered image of the head demonstrates undulating contour and mass-like scalp appearance in the posterior parietal region

Additionally, the patient had an uncomplicated birth history, delivered vaginally at term, with no significant past medical history or use of medication. On examination, scalp bogginess was palpated, and the patient expressed tenderness to palpation around the superior parietal area. The rest of the examination, laboratory tests, and vital signs were unremarkable. Sagittal noncontrast computed tomography of the head, demonstrated in Figure 1, showed an undulating scalp contour in the posterior parietal region. A 3D surface-shaded volume image in Figure 2 demonstrates a mass-like appearance of the scalp in the posterior parietal region. The patient was subsequently discharged after being given headache precautions and supportive care, with plans to follow up with her primary care physician in two weeks.

## Discussion

Subgaleal fluid collections are a rare but important cause of swelling in infants and young children. It typically manifests as an ill-defined, soft, fluctuant, mobile swelling not limited to suture lines [7]. This commonly occurs due to birth trauma, such as prolonged vacuum-assisted delivery, forceps delivery, or a cephalic presentation [8]. It can be diagnosed clinically without radiological imaging, but prompt imaging is required if there is any suspicion about the nature of the swelling or etiology. In recent years, many cases have been seen where this swelling has been caused by trauma to the head due to pulling of hair or aggressive brushing, as in the case of our patient. This condition can commonly resolve spontaneously with no indicated treatment; follow-up is only necessary with the advancement of symptoms [7]. However, it is important to take note and contrast this condition to SGHs, which can occur through the same mechanism as fluid collections but will typically occur in older children from traumatic events [7,8].

SGHs beyond the neonatal period are a rare phenomenon and typically present after significant head trauma. This is commonly due to radial or tangential pulling forces that cause shearing and rupture of the emissary veins that transverse throughout the subgaleal space [9]. The scalp layers can be described using the acronym SCALP, from superficial to deep: skin, connective tissue, aponeurosis, loose areolar tissue, and periosteum [5,10]. SGH occurs in the loose areolar tissue layer, located between the orbital ridge and nuchal ridge anteriorly and posteriorly, respectively, galea aponeurosis superiorly, periosteum inferiorly, and lateral temporal fascia [11]. This phenomenon does not happen as often in adults because, in comparison, adults have relatively few emissary veins, and therefore, sheer force is typically not enough to cause significant damage [12].

At this stage, SGH will present with scalp swelling characterized by crossing the suture lines, and patients can present with sequelae of symptoms, including headache, emesis, amnesia, or drowsiness [5]. Another rare complication occurs when SGH rebleeds. This can cause hematoma expansion of blood into the orbit, face, and neck, leading to visual deficits, proptosis, corneal ulceration, ophthalmoplegia, facial edema, or airway compromise [3,5,13]. A characteristic finding can be the inability to open one's eyes [14].

Optimizing the outcome for children with SGH requires early diagnosis, careful monitoring, and prompt treatment. Nonaccidental head injuries should always be considered in children with spontaneous scalp swelling; SGH has been associated with conditions such as shaken baby syndrome, conditions where there are typically no other supporting physical or historical findings [8]. This was not suspected in our patient. SGH in the absence of a hematological condition is rare, so a thorough evaluation is needed; physicians must be specific about the history of conditions, such as easy or free bleeding and medications the patient may be on [5,9]. In many cases, SGH has been reported as the first manifestation of coagulation disorders, such as factor XIII deficiency and von Willebrand disease [4,15].

SGH treatment is usually conservative; the blood typically will self-resolve within a few weeks [11]. Treatment with aspiration or surgery is generally contraindicated and only used in complicated hematoma cases, such as patients with hemodynamic instability, extended or infected hematoma, or dural sinus injury [5,9]. If none of these are present, conservative treatment has the most efficient approach and outcome, as done with this patient, who recovered uneventfully.

## Conclusions

Subgaleal fluid collections typically occur during birth trauma or from mild head trauma in adolescence. This condition is relatively benign and will resolve itself. It is essential to work with the patient carefully and rule out the more severe phenomenon, SGHs. While SGH is typically rare after the neonatal stage, it can be seen in pediatric and adolescent patients. Workup of these patients should include hematological evaluation and evaluation for child abuse. Trauma to the scalp from hair, including hair pulling or excessively tight braids, can lead to SGH and should be on the differential. Additionally, braids are culturally important to many ethnic groups, so it would not be ethical to advise parents not to braid their children’s hair. However, what can be guided is to recommend these families not to braid their hair too tight because, while rare, there is a risk of subgaleal fluid collections, and rarely SGH and bleeding among young children.
